# *hEGR1* is induced by EGF, inhibited by gefitinib in bladder cell lines and related to EGF receptor levels in bladder tumours

**DOI:** 10.1038/sj.bjc.6603620

**Published:** 2007-02-20

**Authors:** J E Nutt, P A Foster, J K Mellon, J Lunec

**Affiliations:** 1 Northern Institute for Cancer Research, Newcastle University Medical School, Newcastle upon Tyne NE2 4HH, UK; 2 Department of Cancer Studies & Molecular Medicine, University of Leicester, Leicester LE5 4PW, UK

**Keywords:** Gefitinib, EGR1, array analysis, EGF-receptor, bladder tumours

## Abstract

The effect of EGF and gefitinib on two EGFR-positive human bladder cancer cell lines has been investigated using array-based gene expression profiling. The most prominent transcript, increased up to 6.7-fold by EGF compared with controls in RT112 cells, was human early growth response protein 1 *(hEGR1)*. This induction was prevented by gefitinib. The *hEGR1* mRNA in EGF-treated samples was reduced in the presence of gefitinib, as was hEGR1 protein in cell lysates. In the RT4 cells, *hEGR1* expression was halved in the presence of EGF and gefitinib in combination. In bladder tumour samples, there was a significant correlation between *hEGR1* mRNA detected by RT-PCR and EGFR detected by ligand binding, (*P*=0.042). The induction by EGF of the *hEGR1* gene, mRNA and protein in RT112 cells, and its inhibition by gefitinib, together with the detection of *hEGR1* mRNA in bladder tumours, suggests that *hEGR1* may be important in the EGFR growth-signalling pathway in bladder cancer and should be further investigated for its prognostic significance and as a potential therapeutic target.

Growth factors and their receptors are important in tumour development and progression. Several studies have shown that the presence of epidermal growth factor receptor (EGFR) in bladder cancer is associated with high tumour stage and grade and is a strong independent predictor of tumour stage progression and poor long-term survival (Neal *et al*, 1985, 1990; Lipponen and Eskelinen, 1994; Mellon *et al*, 1995). Bladder cancer is the fifth most common cancer in men, with an annual incidence of 24.7 per 100 000 population in the UK. 90% of bladder tumours are transitional cell carcinoma (TCC) and can be broadly divided into superficial (Ta and T1) or muscle invasive (T2, T3 and T4) forms. Approximately 50–70% of superficial tumours recur, following removal at cystoscopy, of which 10–20% will become invasive. At present there is no reliable method to predict which superficial tumours will show invasive progression or metastasize.

EGFR is a member of the erbB family of cell surface receptors, which comprises four homologous receptors: EGFR (erbB-1/HER1); erbB-2 (HER2/neu); erbB-3 (HER3) and erbB-4 (HER4). These receptors are composed of an extracellular ligand-binding domain, a transmembrane domain and an intracellular protein kinase domain. Ligand binding activates the EGFR by inducing homo- or hetero-dimerisation with other members of the erbB family, resulting in autophosphorylation of both intracellular tyrosine kinase domains. This initiates a cascade of intracellular signalling events that promote growth and the expression of genes involved in malignant progression (Olayioye *et al*, 2000).

EGFR signalling is critical not only for cell proliferation but also in other processes crucial to cancer progression, and EGFR has therefore been explored as a target for anticancer therapies. One approach for the therapeutic blockade of EGFR signalling has been the discovery and development of low molecular weight compounds that inhibit the ligand-binding induced activation of the EGFR tyrosine kinase. Gefitinib (‘Iressa’, ZD1839) is an orally active EGFR tyrosine kinase inhibitor (EGFR-TKI). It is a low molecular weight synthetic quinazoline derivative that has been shown to block signal transduction pathways implicated in the proliferation and survival of cancer cells and other host-dependent processes promoting cancer cell growth (Wakeling *et al*, 1996; Woodburn, 1999). Gefitinib and other EGFR inhibitors, and results of clinical trials using gefitinib in cancer patients, have recently been reviewed (Blackledge and Averbuch, 2004; Herbst *et al*, 2004).

This study investigates the effect of the tyrosine kinase inhibitor gefitinib and EGF on the gene expression patterns of two human bladder tumour cell lines, and the subsequent investigation of human early growth response protein 1 (hEGR1) found to be prominently induced by EGF but not in the presence of gefitinib. The relationship between hEGR1 and EGFR in human bladder tumours was also investigated.

## METHODS

### Cell culture

Two human bladder tumour cell lines, RT4 and RT112, were obtained from ECACC (Porton Down, UK) and routinely grown in RPMI 1640 medium containing L-glutamine, 1 mM pyruvate and 10% foetal bovine serum (FBS). All cells were negative for mycoplasma. Both cell lines expressed EGFR (Nutt *et al*, 2004) and cells were treated as described previously (Nutt *et al*, 1998). Briefly, cells at 50% confluence were depleted of serum for 24 h to remove exogenous growth factors and treated with 10 ng ml^−1^ EGF (Sigma-Aldrich) and/or 1 *μ*M gefitinib in serum-free medium continuously for 48 h. Gefitinib (1 *μ*M) had previously been shown not to inhibit cell growth (Nutt *et al*, 2004). Following incubation, the cells were washed twice with ice-cold PBS, scraped from the culture dishes, centrifuged and the cell pellets stored at −80°C prior to extraction of RNA. For Northern analysis, cells were treated with 10 or 50 ng ml^−1^ EGF for up to 48 h and processed as above. Control cells were grown in exactly the same way in serum-free medium.

### Array analysis

RNA was extracted using Qiagen RNeasy Midi kit, the lysis buffer being added directly to the frozen cell pellets, which were then homogenised by 10–15 passes through a 20 gauge needle. RNA samples were treated with DNase (Ambion DNA free kit) and the RNA quality was assessed using 1.2% agarose gel electrophoresis with ethidium bromide staining. RNA concentrations were measured using absorbance at 260 nm.

Gene expression analysis was performed using Clontech Atlas Human Cancer 1.2 Arrays (BD Biosciences, Cowley, Oxford, UK). cDNA labelled with ^32^P was synthesised from the samples of total RNA using the Clontech Pure Total RNA labelling kit (the RNA is immobilised onto streptavidin-coated magnetic beads with biotinylated oligo(dT) and allows both poly A^+^ RNA enrichment and probe synthesis in a single procedure). The RNA was reverse transcribed with a specific primer mix for the genes on the array. The cDNA mixture was then separated from unincorporated labelled nucleotides using nucleospin extraction columns supplied in the kit. The filters were prehybridised at 68°C with denatured salmon testes DNA, and hybridised with Cot-1 DNA (GibcoBRL, Life Technologies, Invitrogen Ltd, Paisley, UK) as blocking agents on the filters. Hybridisation was performed overnight at 68°C. The array filters were washed six times in 2 × SSC/1%SDS buffer, prior to exposure to a Molecular Dynamics Phosphor screen for 1–5 days at 4°C.

Signals were analysed using Molecular Dynamics ImageQuant v5.0 and Clontech AtlasImage Analysis v1.5 and normalised using the sum of global intensities. The intensity difference and expression ratio were calculated for each gene. The expression ratio was defined as the intensity of the gene signal in the treated sample compared with the serum-free control.

After analysis, the array membranes were stripped by boiling in 0.5% SDS for 5 min. Repeat hybridisations were performed with freshly prepared probes and cross over of samples. Membranes were reprobed a maximum of three times.

### PCR analysis

To confirm the *hEGR1* gene expression and its modulation by EGF and gefitinib in the cell lines, RT-PCR was performed using the Invitrogen Superscript First strand synthesis system with random primers. The human *EGR1* specific primers used were: SN CTGCACGCTTCTCAGTGTTC and ASN AGCAGCATCATCTCCTCCAG (Chen *et al*, 2001). PCR was performed for 36 cycles: 94°C 30 s; 55°C 30 s; 72°C 30 s; then 4°C. PCR products were analysed by electrophoresis on a 2% NuSieve 3:1 (Flowgen Bioscience Ltd, Nottingham, UK) agarose gel and detected by fluorescence staining with ethidium bromide.

### Western blot analysis

Western blotting was performed to determine the effects of EGF on expression of the hEGR1 protein. Cell lysates were prepared and 60 *μ*g aliquots of protein from each sample were loaded on a 4–20% gradient polyacrylamide gel. Following SDS-PAGE and electroblotting (Towbin *et al*, 1979), membranes were blocked using a 5% milk solution prior to incubation with an hEGR1 rabbit polyclonal antibody (Santa Cruz, sc-110) at a final concentration of 1 *μ*g ml^−1^ followed by a secondary HRP-conjugated antibody (Goat-antirabbit, Dako UK Ltd, Ely, Cambridgeshire, UK). Antibody labelled protein bands were visualised by enhanced chemiluminescent detection (ECL) (Amersham Biosciences, GE Healthcare UK Ltd, Little Chalfont, Bucks, UK). Equal loading of protein was verified using anti-actin antibody (Sigma-Aldrich Co Ltd, Poole, UK).

### RNA extraction and Northern blot analysis

Total RNA was extracted from the frozen cell pellets using the phenol-guanidinium isothiocyanate method (Chomczynski and Sacchi, 1987) with the commercial reagent RNAzol (Biogenesis, Poole, UK). The RNA pellet was dissolved in water, the concentration determined by absorbance at 260 nm and the samples stored at −70°C.

Northern blot analysis was performed using the glyoxal method as previously described (Nutt *et al*, 1991). Briefly, 20 *μ*g samples of RNA were treated by glyoxylation prior to electrophoresis on 1.2% agarose gel in 10 mM phosphate buffer. The gel was stained with ethidium bromide to verify equal loading of samples before capillary transfer of the RNA overnight onto Hybond-N nylon membrane (Amersham Biosciences, Little Chalfont, UK). After transfer and air drying, RNA was fixed to the membrane by ultra violet irradiation cross-linking for 3.5 min using a mid range transilluminator (UltraViolet products Inc., UK).

Radioactive probes, from cDNA prepared by RT-PCR, were prepared by the random primer extension method using *α*^32^P-ATP (Amersham, UK). Primers for *hEGR1* probe preparation were designed to give a product of approximately 1 kb: SN 5′-CTTCAACCCTCAGGCGGACACG-3′; ASN 5′-CGGGGACGGGTAA GAGGTAGCA-3′. RNA samples were used for RT-PCR, using the Invitrogen Superscript First strand synthesis system. PCR products were electrophoresed in a 1% agarose gel and the product detected and yield estimated by ethidium bromide staining. The product band was extracted using a QIAquick gel extraction kit (Qiagen, UK). DNA (50 ng) aliquots were used directly in the random primer extension radioactive labelling reaction.

After deglyoxylation by boiling in water for 5 min, the filters were prehybridised at 65°C for 3 h in hybridisation solution (0.5 M sodium phosphate buffer, pH 7.0; 1 mM EDTA; 1% BSA; 7% SDS) with 1 *μ*g ml^−1^ denatured salmon sperm DNA, to block non-specific DNA binding sites. Hybridisation was carried out overnight using 10^6^ cpm of probe ml^−1^ hybridisation solution. The filters were then washed twice in 2 × SSC, 0.2% SDS at 65°C for 5 min and a final wash for 15 min at 65°C. To detect the bound radioactivity the filters were exposed to a PhosphorImager screen (Molecular Dynamics, UK).

### PCR analysis of bladder tumours

RNA prepared from human bladder tumours was reverse transcribed as previously described. PCR was performed for both *hEGR1* and *actin*. The primers used for *actin* were SN 5′-CAA CTC CAT GAA GTG TGA-3′ and ASN 5′-GCC ATG CCA ATC TCA TCT TG-3′ to give a product size of 377 bp. The primers for *hEGR1* and *actin* were used in the ratio 4 : 1. PCR was performed by heating to 94°C for 10 min, then 40 cycles of 94°C 30 s, 55°C 30 s, 72°C 1 min, followed by 72°C 7 min, then 4°C.

PCR products were run on an agarose gel and bands stained with ethidium bromide were quantified using a Biorad Imager. Samples from RT112 and RT4 cells treated with 10 ng ml^−1^ EGF for up to 4 h were also used, and the RT112 sample treated for 1 h was used as a positive control in all runs. The ratio of *hEGR*/*actin* from densitometry was compared with the tumour EGFR level obtained by ligand binding assay as previously reported for this panel of tumours, with EGFR positive tumours defined by EGF binding of >10 fmol mg^−1^ protein (Mellon *et al*, 1996).

## RESULTS

### Array analysis

The Qiagen kit yielded high quality, pure RNA and agarose gel electrophoresis consistently showed no degradation of the RNA. The array analysis produced well-defined expression profiles with low background intensity.

**Figure 1:**
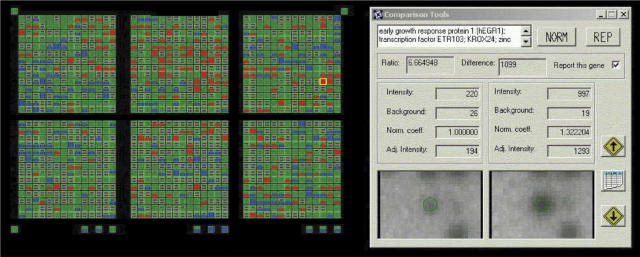
AtlasImage comparison between array hybridisations showing differential expression of *hEGR1* in EGF-treated RT112 cells. The intensity is calculated for cells treated with 10 ng ml^−1^ EGF (right) and the serum free controls (left) and the ratio between treated and untreated controls is calculated from the adjusted intensities for each gene.

In the RT112 cell line, the most prominent increase in expression was that of the *hEGR1* gene which was induced in multiple hybridisations when the cells were stimulated with 10 ng ml^−1^ EGF. In four hybridisations from two cell culture experiments, *hEGR1* was clearly visible above background on control arrays. *hEGR1* was induced by EGF, with expression ratios ranging from 2.3 to 6.7 and a mean expression ratio of 4.7 was obtained from three experiments. A comparison of hybridisation to the array between cDNA probes made from control and EGF treated cells showing differential expression of *hEGR1* in RT112 cells treated with EGF is shown in Figure 1. This illustrates an example of the reproducibility observed. The expression of *hEGR1* was suppressed with 1 *μ*M gefitinib, giving an expression ratio of 0.5 when the treated samples were compared with controls. When both 10 ng ml^−1^ EGF and 1 *μ*M gefitinib were incubated together with the cells, an expression ratio of 0.56 was obtained.

With the RT4 cell line, *hEGR1* was again induced when the cells were incubated with EGF and with gefitinib. Although the intensity of *hEGR1* was increased the expression ratio was undefined, since a ratio requires above background gene expression in membranes used with both control and treated samples. However, EGF and gefitinib used in combination was observed to suppress the *hEGR1* seen as above background in the untreated control sample. The expression ratio with the combined treatment ranged from 0.26 to 0.5.

Some of the other genes affected by EGF treatment are listed in Table 1 (RT112 cells) and Table 2 (RT4 cells). Genes with the highest ratio for upregulation and the lowest ratio for down regulation with EGF treatment are included. *hEGR1* was the only gene where differential expression was observed between EGF and combined EGF/gefitinib treatment in both cell lines.

### RT-PCR validation of *hEGR1* induction

**Figure 2:**
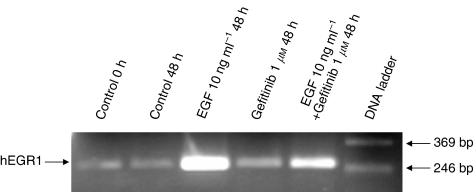
RT112 cell RT-PCR products detected with ethidium bromide following electrophoresis in a 2% agarose gel. Treatment of cells was for 48 h as indicated.

RNA samples from RT112 cells were used for RT-PCR using primers for *hEGR1* designed to give a product of 253 base pairs (see Methods). The results are shown in Figure 2, with EGF showing a large increase in the *hEGR1* RT-PCR product compared to the control. Very little product was present with gefitinib alone, and the product with EGF and gefitinib in combination was reduced compared with EGF alone, confirming the inhibitory effect of gefitinib on the induction of *hEGR1* transcripts by EGF.

### Northern analysis

**Figure 3:**
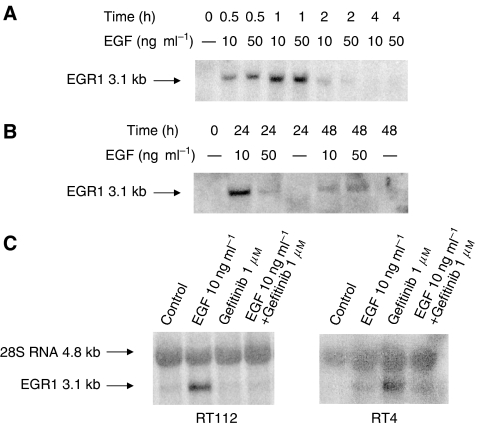
Northern blot analysis of RNA using a radioactive probe for *hEGR1*. (**A**) Early induction of *hEGR1* following EGF stimulation in RT112 cells; (**B**) sustained *hEGR1* induction following EGF stimulation for 24 and 48 h in RT112 cells; (**C**) induction of *hEGR1* by EGF at 48 h and inhibition of this induction with gefitinib in RT112 (left panel) and RT4 cells (right panel). 28S RNA bands indicate equal loading of RNA.

The time course of *hEGR1* mRNA induction following EGF treatment of RT112 cells was investigated by Northern blot analysis (Figure 3A and B). A single 3.1Kb transcript was detected. *hEGR1* transcript levels increased rapidly within 30 min of EGF treatment, with both 10 and 50 ng ml^−1^ EGF, and reached a maximum at 1 h. After 2 h continuous treatment, transcript levels had fallen, and by 4 h there was no evidence of any *hEGR1* induction (Figure 3A). Later time points at both 24 and 48 h showed induction with EGF, with no evidence of induction of *hEGR1* in controls without EGF grown in serum-free medium for the same length of time (Figure 3B).

Incubation of RT112 cells for 48 h with 1 *μ*M gefitinib alone showed no *hEGR1* induction. The increase in levels of *hEGR1* mRNA seen with EGF treatment was prevented by the presence of gefitinib (Figure 3C). Equiloading was demonstrated by the 28S RNA band. These results demonstrate that *hEGR1* is induced as an early growth response protein, but is also elevated at later time points and that the induction by EGF is inhibited by the EGFR tyrosine kinase inhibitor.

In the RT4 cells, induction of *hEGR1* was again seen when cells were treated with EGF for 48 h (Figure 3C) in agreement with the results from the array analysis.

### Western analysis

**Figure 4:**
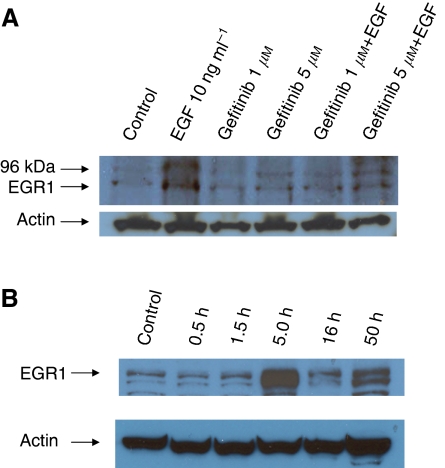
Western blot analysis for hEGR1 and actin in RT112 cell lysates following treatment of cells: (**A**) with EGF, gefitinib or a combination for 48 h; (**B**) with EGF for up to 50 h.

Western Analysis for hEGR1 protein in the RT112 and RT4 cell lysates treated with EGF with or without gefitinib for 48 h, detected very low levels of hEGR1 protein following treatment only with EGF alone and not in the presence of gefitinib (Figure 4A). Subsequently, hEGR1 protein was detected at 80 kDa following EGF stimulation with 10 ng ml^−1^ at 5 and 50 h in the RT112 cell lysate (Figure 4B). The gels were also probed for *β*-actin, shown in the lower panel of each part of the figure.

### PCR analysis of bladder tumours

**Figure 5:**
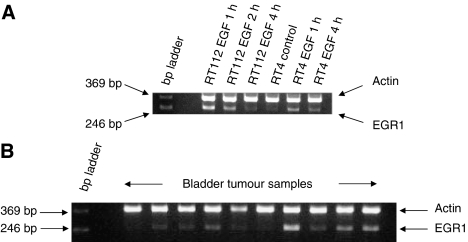
Agarose gel electrophoresis of RT-PCR products of *hEGR1* and *actin* (**A**) from RT112 and RT4 cells treated with EGF; (**B**) from a selection of bladder tumour samples.

**Figure 6:**
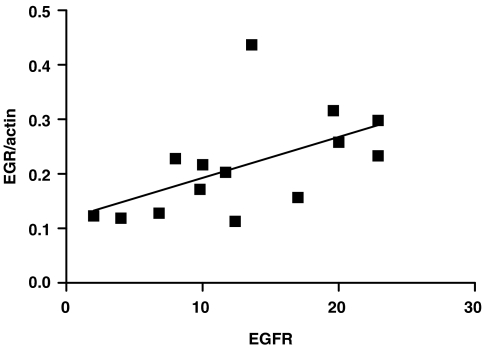
Relationship between *hEGR/Actin* ratio and EGFR (fmol mg^−1^ protein) measured by ligand binding.

By using RT-PCR, *hEGR1* was shown to be induced in both RT112 and RT4 cells following stimulation with EGF after 1 h. The induction was reduced to control levels after 4 h of treatment (Figure 5A). The RT112-treated samples were used as a positive control in subsequent analyses of bladder tumour samples and using densitometry gave a mean ratio of *hEGR/actin* of 0.54 (s.d.±0.11, *n*=3). A selection of the bladder tumour samples analysed is shown in Figure 5B. The ratio of *hEGR/actin* ranged from 0.113 to 0.437. The results from triplicate experiments for 14 bladder tumour samples of known and previously reported EGFR status approached significance at the 95% confidence level (*P*=0.054, Mann–Whitney test), with a trend towards higher *hEGR/actin* ratios in EGFR positive tumours. A tumour was defined as EGFR positive if the ligand binding was >10 fmol mg^−1^ protein and showed positive staining for EGFR by immunohistochemistry. The mean values (±s.e.m.) of the EGFR positive samples and EGFR negative samples were 0.252 (±0.036) and 0.165 (±0.020) respectively. There was, however, a significant correlation between the EGFR measurements from ligand binding assays and the *hEGR/actin* ratio (*P*=0.042, *r*^2^=0.302) as shown in Figure 6. There was no statistical difference in *hEGR/Actin* ratio in relation to stage or grade of tumours (data not shown).

## DISCUSSION

Intracellular signalling from the EGF receptor is known to follow two pathways, the activated ras and mitogen-activated protein (MAP) kinase cascade or the JAK-STAT (janus kinase/signal transducers and activators of transcription) complex (Leaman *et al*, 1996). The involvement of hEGR1 in the EGFR stimulation of bladder tumour cells adds further detail to the downstream effects of intracellular signalling from EGFR in bladder tumours. Several immediate early response genes are transiently activated in serum or growth factor stimulated cells in culture and include the early growth response protein-1 (*hEGR1*)/nerve growth factor-induced protein A (*NGFI-A*). EGR1 is a nuclear zinc-finger transcription factor belonging to the EGR family, which includes WT1. Extracellular stimuli that induce EGR1 can be grouped into mitogens, developmental and differentiation processes, tissue or radiation injury and signals that cause neuronal excitation (Gashler and Sukhatme, 1995). EGR1 can act as a positive or negative regulator of gene expression, depending on cell type, although its precise function in growth regulation remains unclear.

There have been few reported studies of hEGR1 in bladder cancer but the role of hEGR1 in prostate tumours has recently been reviewed (Adamson *et al*, 2003). *hEGR1* expression has been reported to correlate with increased growth and malignancy in prostate cancer, and to be involved in the maintenance of prostate tumour cell proliferation (Thigpen *et al*, 1996; Eid *et al*, 1998). In the latter study, progression of tumours directly required EGR1, and was halted in studies involving *EGR1*−/− knockouts. Using gene microarrays, expression of hEGR1 target genes in prostate carcinoma cells were identified and include growth factors such as IGFII, PDGF-A and TGF-*β*1 which have been implicated in enhancing tumour progression (Svaren *et al*, 2000). Increased levels of *hEGR1* mRNA were demonstrated in poorly differentiated malignant prostate tissues and those with more aggressive pathology (Eid *et al*, 1998). As RT112 cells were derived from a moderately differentiated tumour and RT4 cells from a well differentiated tumour (Masters *et al*, 1986), this could explain the larger *hEGR1* induction in the RT112 cell line. The RT112 cells are also more invasive than the RT4 cells (Booth *et al*, 1997), and hence more aggressive, which may also affect the *hEGR1* induction.

Most reports on EGR1 demonstrate the gene product to be rapidly and transiently expressed and *EGR1* is generally seen as an early response gene (with increased levels usually within an hour of cellular stimulation). Our results demonstrate not only an early (1–2 h) transient response to EGF, but also a subsequent prolonged response at both 24 and 48 h in RT112 cells. This response is seen at both the mRNA and protein level, and is inhibited by the EGFR tyrosine kinase inhibitor gefitinib. It is possible that most previous investigations of EGR1 induction have only been studied over a short time scale, as the name would suggest. Also, since *hEGR1* is induced during the G_0_ to G_1_ transition (Molnar *et al*, 1994) and the doubling time for the RT112 cells is approximately 24 h (Masters *et al*, 1986) this may explain the induction of *hEGR1* at the later time points. It could also be due to the auto-induction of EGFR (Saadane *et al*, 2000). EGFR expression was upregulated after induction of *hEGR1* during hypoxic exposure of human tumour cells (U-2OS) (Nishi *et al*, 2002) and it was found that hEGR1 directly induced EGFR transcription. This positive autoregulatory feedback loop that restores EGFR protein levels to enable tumour growth in hypoxic conditions may also occur in other conditions. A study of the expression of *hEGR1* in late stage emphysema (Zhang *et al*, 2000) demonstrates that lung tissue from these patients compared with control samples displays a selective and apparently sustained increase in *hEGR1* transcripts and proteins compared with a broad survey of other genes, including the transcription factor SP1 which was not significantly altered. The sustained *hEGR1* induction described leads to the question as to whether hEGR1 and hEGR1-regulated genes contribute to the pathogenesis or progression of late stage emphysema. It is interesting that, when induced, hEGR1 often displaces other zinc finger transcription factors such as SP1 from the GC-rich binding sites where the transcription factors support basal expression of genes such as tissue factor (TF) resulting in maximal transcriptional activation of the target genes (Cui *et al*, 1996).

The expression of hEGR1 in normal or malignant tissues varies, depending on the tissue. *hEGR1* expression was reported not to be significantly higher in bladder cancer than normal tissue (Ogishima *et al*, 2005) and no significant relationship was observed between *hEGR1* expression and pathological findings. Our results demonstrate a significant correlation between the number of EGF receptors and the expression of *hEGR1* in bladder tumours, although no difference in *hEGR1* expression was detected with stage or grade of tumour. Increased levels of hEGR1 have been demonstrated in prostate cancer compared with benign prostate tissue, with hEGR1 located predominantly in the cytoplasm in malignant cells (Mora *et al*, 2004). In breast cancer cell lines and tumours, reduced hEGR1 expression compared with normal mammary cell lines and tissues has been reported (Huang *et al*, 1997). The authors suggested that *hEGR1* may act like a tumour suppressor gene of the Type II class where expression is down regulated in tumours. *hEGR1* could therefore be a target for therapy by reactivating its expression in this situation. Little or no *hEGR1* expression was found in human small cell lung tumours compared with adjacent normal tissues (Levin *et al*, 1995). From studies using *EGR1*-null mouse embryo fibroblasts it was proposed that *EGR1* is a major regulator of cell senescence and an upstream ‘gatekeeper’ of the p53 tumour suppressor pathway (Krones-Herzig *et al*, 2003).

A study on human gliomas (Calogero *et al*, 2001) also suggests that *hEGR1* may function as a tumour suppressor. hEGR1 was found to be suppressed at both mRNA and protein levels in gliomas and hEGR1 itself is implicated as a target of suppression during tumour progression. Studies on glioma cells (Kaufmann and Thiel, 2001) following EGF stimulation indicated that hEGR1 may be an important part of the EGF-initiated signalling cascades and showed that *hEGR1* gene transcripts and protein were undetectable in the absence of cell stimulation by EGF. A similar proposal that hEGR1 may be an important late part of the EGF signalling cascade is reported in a study of EGF and thrombin on keratinocytes (Kaufmann and Thiel, 2002). EGF induced a transient synthesis of hEGR1 with a peak between 1 and 5 h. Proliferation, activation of ERK and biosynthesis of hEGR1 was inhibited by AG1487, an EGFR specific tyrosine kinase inhibitor. Another tyrosine kinase inhibitor, erlotinib, has been shown to inhibit a potent radiation-induced enhancement of hEGR1 in a head and neck SCC cell line (UM-SCC6) using a 24 h pre-incubation with erlotinib (Chinnaiyan *et al*, 2005). Our results demonstrate the inhibition of *hEGR1* induction in bladder tumour cell lines with the EGFR tyrosine kinase inhibitor gefitinib.

Other genes found to be affected by EGF and gefitinib have not been further investigated since *hEGR1* was the only gene which was upregulated with EGF and downregulated with a combination of EGF and gefitinib treatment in both cell lines. It is, however, interesting that EGF produced opposite effects on tissue inhibitor of metalloproteinase (TIMP) 3 and TIMP1 in both cell lines and only a few genes were seen to be affected in the same way in the two cell lines studied (upregulation of *c-jun* and *TIMP3*; downregulation of *TIMP1*).

The sustained induction of *hEGR1* by EGF in the RT112 bladder tumour cells for 24 and 48 h, in addition to the rapid induction seen at 1 to 2 h, indicates that further investigation of the role of hEGR1 and EGFR in bladder tumours is warranted. The presence of *hEGR1* in tumours is shown here to be related to EGFR status and may be an important causal link to the growth and progression of bladder tumours. The detection of *hEGR1* mRNA in varying quantities in all tumour samples used in this study and the significant correlation between *hEGR1* and EGFR are an indication of its importance in bladder cancer. To date there are no reports as to whether there are any somatic mutations in *EGFR* in bladder cancer cell lines which may affect the responsiveness to gefitinib (Lynch *et al*, 2004). The absence of *hEGR1* mRNA in the bladder tumour cells without EGF stimulation also indicates that *hEGR1* is not constitutively expressed. Further investigation of the intracellular signalling pathways involved in the *hEGR1* induction by EGF and its inhibition by gefitinib would clarify the role of *hEGR1* in EGFR-dependent tumour growth and progression and its inhibition by gefitinib.

## Figures and Tables

**Table 1:** Gene transcript levels altered by EGF in RT112 cells and effects of gefitinib treatment with and without EGF

**Gene**	**EGF**	**Gefitinib**	**EGF+gefitinib**
Cytosolic superoxide dismutase 1	**6.9**	**6.6**	**4.8**
Vitronectin precursor	**5.3**	ND	ND
Early growth response protein 1	**4.7**	0.5	0.56
Interferon *γ*-induced protein precursor	**4.7**	**2.5**	ND
c-jun	**4.0**	**3.4**	**3.9**
Tissue inhibitor of metalloproteinase 3	**4.0**	ND	**2.4**
			
Cytohesin-1	0.2	ND	ND
Tissue inhibibitor of metalloproteinase 1	0.2	0.45	0.3
GTP-binding protein	0.3	ND	ND
Alpha 1 catenin	0.3	0.4	ND
Cytokeratin 8	0.3	ND	ND
BRCA1 associated ring domain protein	0.35	0.4	0.35

Numbers indicate the ratio of expression in treated cells relative to untreated controls. Bold indicates upregulation of the gene compared to control treatment. ND indicates there is no difference relative to the untreated control.

**Table 2:** Gene transcript levels altered by EGF in RT4 cells and effects of gefitinib treatment with and without EGF

**Gene**	**EGF**	**Gefitinib**	**EGF+Gefitinib**
Integrin *β*4	**4.1**	ND	0.2
Early growth response protein 1	**Un >3**	**Un >3**	0.38
P16-INK4	**3.0**	**4**	0.2
Macrophage inhibitory cytokine1	**3.0**	ND	**1.2**
Interferon-induced protein P78	**3.0**	ND	ND
c-jun	**2.6**	ND	ND
Tissue inhibitor of metalloproteinase 3	**2.6**	ND	ND
			
Semaphorin	0.2	0.3	ND
Cytokeratin 18	0.2	0.3	0.1
Tissue inhibitor of metalloproteinase 1	0.4	0.5	0.35
Fatty acid synthase	0.4	ND	ND
Lactate dehydrogenase H subunit	0.4	ND	**2.2**

Numbers indicate the ratio of expression in treated cells relative to untreated controls. Bold indicates upregulation of the gene compared to control treatment. ND indicates there is no difference relative to the untreated control. **Un** indicates the ratio was undefined because the untreated control levels were below background.
